# Polyaromatic *Bis*(indolyl)methane Derivatives with Antiproliferative and Antiparasitic Activity

**DOI:** 10.3390/molecules28237728

**Published:** 2023-11-23

**Authors:** Raquel C. R. Gonçalves, Pablo Peñalver, Susana P. G. Costa, Juan C. Morales, Maria Manuela M. Raposo

**Affiliations:** 1 Centre of Chemistry, University of Minho, Campus of Gualtar, 4710-057 Braga, Portugal; raquelrainha10@hotmail.com (R.C.R.G.); spc@quimica.uminho.pt (S.P.G.C.); 2 Advanced (Magnetic) Theranostic Nanostructures Lab, International Iberian Nanotechnology Laboratory, Av. Mestre José Veiga s/n, 4715-330 Braga, Portugal; 3 Instituto de Parasitología y Biomedicina López Neyra, CSIC, PTS Granada, Avenida del Conocimiento 17, 18016 Armilla, Granada, Spain; pablo@ipb.csic.es (P.P.); jcmorales@ipb.csic.es (J.C.M.)

**Keywords:** African trypanosomiasis, antiproliferative agent, antiprotozoal agent, *bis*(indolyl)methane derivatives, leishmaniasis

## Abstract

*Bis*(indolyl)methanes (BIMs) are a class of compounds that have been recognized as an important core in the design of drugs with important pharmacological properties, such as promising anticancer and antiparasitic activities. Here, we explored the biological activity of the BIM core functionalized with different (hetero)aromatic moieties. We synthesized substituted BIM derivatives with triphenylamine, *N,N*-dimethyl-1-naphthylamine and 8-hydroxylquinolyl groups, studied their photophysical properties and evaluated their in vitro antiproliferative and antiparasitic activities. The triphenylamine BIM derivative **2a** displayed an IC_50_ of 3.21, 3.30 and 3.93 μM against *Trypanosoma brucei*, *Leishmania major* and HT-29 cancer cell line, respectively. The selectivity index demonstrated that compound **2a** was up to eight-fold more active against the parasites and HT-29 than against the healthy cell line MRC-5. Fluorescence microscopy studies with MRC-5 cells and *T. brucei* parasites incubated with derivative **2a** indicate that the compound seems to accumulate in the cell’s mitochondria and in the parasite’s nucleus. In conclusion, the BIM scaffold functionalized with the triphenylamine moiety proved to be the most promising antiparasitic and anticancer agent of this series.

## 1. Introduction

Pathogenic protozoa are responsible for several diseases worldwide including human African trypanosomiasis (also known as sleeping sickness, caused by two subspecies of *Trypanosoma brucei*) and leishmaniasis (caused by more than 20 species of *Leishmania* spp.). Currently, these parasitic diseases do not have a FDA-approved vaccine [1,2,3,4], and the drugs used as treatments have many drawbacks such as toxicity, undesirable side effects, drug resistance, conditional efficiency (depending on the species causing the infection and the stage of disease) and high cost. In 2021, the US Food and Drug Administration (FDA) approved fexinidazole as the first all-oral treatment for both stages of the *Trypanosoma brucei gambiense* form of sleeping sickness [5]. Nevertheless, additional therapeutic options are needed for this parasitic infection, and especially for other tropical neglected diseases.

**Figure 1:**
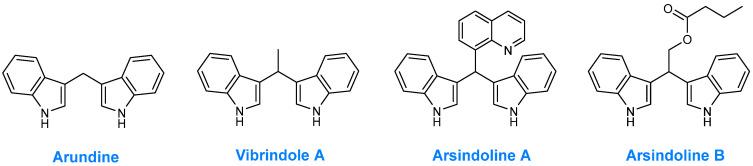
Structures of arundine, vibrindole A, arsindoline A and arsindoline B.

*Bis*(indolyl)methanes (BIM) are a class of heterocyclic compounds that have as their central core two indole units linked through a methylene group. This structure can be found as part of several natural products including arundine, vibrindole A, arsindoline A and arsindoline B (see Figure 1) [6,7,8]. BIM derivatives have been already shown to have interesting pharmacological activities such as anticancer [9,10,11,12], antibacterial [13,14], antioxidant [15,16], anti-inflammatory [17,18], antifungal [19] and antiviral agents [20], among others. Indeed, the BIM core is recognized as an important scaffold and pharmacological intermediate in drug discovery; therefore, great effort has been devoted to the synthesis and chemical modification of *bis*(indolyl)methane and its analogues to gain further insight regarding the structure–activity relationship [21].

The antitumor activity and mechanism of action of *bis*(indolyl)methane and its derivatives have been widely investigated in several cancer cell lines [22,23,24,25,26,27]. However, to the best of our knowledge, research on the antiparasitic activity of this family of compounds has been scarce [28,29,30,31,32]. Most notably, Roy et al. reported that *bis*(indolyl)methane was active against *Leishmania donovani* (IC_50_ of 1.2 μM) through a mechanism of action that inhibits topoisomerase I and mitochondrial F0F1-ATP synthase [30,31]. Bharate et al. studied the antileishmanial, antimalarial, antibacterial and antifungal activities of different BIM derivatives. The structure–activity relationship analysis showed that *bis*(indolyl)methane functionalized with nitroaryl or heteroaromatic groups displayed promising antileishmanial activity (IC_50_ ranging from 3.02 to 15.94 μM), although the therapeutic window toward healthy cell lines was not reported [29]. Furthermore, the synergistic interaction of several *bis*(indolyl)methane-related indoles and other known antiparasitic drugs for the treatment and prevention of parasitic infections including malaria, leishmaniasis, trypanosomiasis, trichomoniasis, neosporosis and coccidiosis were protected under patent [33].

Drugs with dual anticancer and antiparasitic activities have been described in the literature. In fact, they possibly target related metabolic routes in tumor cells and parasites as well as other common therapeutic targets [34,35]. For example, carbohydrate naphthalene diimide conjugates (carb-NDIs) and stiff-stilbene derivatives have been reported as promising anticancer and antiparasitic agents whose mechanism of action is associated with high-affinity binding to nucleic G-quadruplex structures [36,37]. Moreover, other possible mechanisms of action of these dual drugs have been comprehensively reviewed and discussed by Zahra Dorosti et al. [34]. Yet, further research in this field is necessary.

Having this in mind, our goal was to design, synthesize and evaluate the biological activity of a family of BIM derivatives **2a**–**c** functionalized with different hetero(aromatic) groups, including triphenylamine, *N,N*-dimethyl-1-naphthylamine and 8-hydroxylquinolyl. Here, we describe the synthesis of compounds **2a**–**c**, as well as the characterization of the structural and photophysical properties and evaluation of their biological activity as potential antiparasitic agents, specifically active against *T. brucei* and *L. major*. Additionally, due to the anticancer properties of previously reported BIM derivatives, the antiproliferative activity and selectivity of the synthesized compounds were also investigated in human cancer cell lines (endometrium and breast cancer cell lines) and compared to their activity in a healthy human cell line (fibroblasts).

## 2. Results and Discussion

### 2.1. Synthesis and Characterization of Bis(indolyl)methane Derivatives

**Scheme 1:**
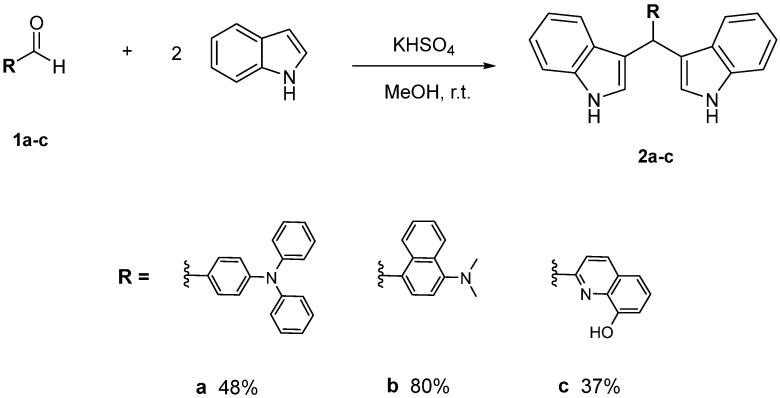
Synthesis of *bis*(indolyl)methane derivatives **2a**–**c**.

A series of *bis*(indolyl)methane derivatives **2a**–**c** were synthesized, purified and characterized in order to evaluate phenotypically the influence of the different (hetero)aromatic moieties on in vitro biological models. As an overview, the route for the synthesis of the BIM derivatives substituted with triphenylamine, *N*,*N*-dimethyl-1-naphthylamine and 8-hydroxylquinolyl groups, is represented in Scheme 1, and the experimental data concerning the synthesis and the structural and photophysical characterization of the compounds are shown in Table 1 and Figures S1–S6.

The compounds were obtained in moderate to good yields (37–80%) through the condensation of indoles with the formyl precursors **1a**–**c** in dry methanol and in the presence of potassium hydrogen sulfate [38]. The synthesis of BIM derivative **2a** has been reported previously [39,40].

The photophysical properties of the *bis*(indolyl)methanes **2a**–**c** were investigated in acetonitrile solutions, and it was observed that the position of the absorption and emission bands were clearly dependent on the nature of the substituent group introduced in the *bis*(indolyl)methane core. Compound **2a** displayed the highest maximum absorption wavelength and the lowest maximum emission wavelength, resulting in a smaller Stokes shift (Δ_SS_ = 7365 cm^−1^). Compound **2b** presented an absorption band at a lower wavelength (**λ_abs_** = 218 nm), while compound **2c** exhibited the highest emission wavelength and the largest Stokes shift (Δ_SS_ = 20790 cm^−1^). Regarding the relative fluorescence quantum yields (*ϕ_F_*), derivative **2a** showed modest fluorescence emission efficiency, whereas derivatives **2b**,**c** were much less fluorescent.

In fact, the position of the absorption bands were clearly dependent on the electronic nature of the substituent group: compound **2a** displayed the highest wavelength of maximum absorption, due to the extended intramolecular electron delocalization of the triphenylamine group as well as its electron-donating nature, and both compound **2b** and **2c**, bearing a naphthalene and quinoline moiety, respectively, showed blue-shifted absorption bands when compared to compound **2a**, probably due to their electron-deficient heterocyclic nature as well as the smaller size of the π-conjugated system.

### 2.2. Biological Phenotypic Activity

The biological activity of the *bis*(indolyl)methane derivatives **2a**–**c** were evaluated on in vitro models of parasites (*T. brucei* and *L. major*), human cancer cells (HeLa and HT-29) and human healthy cells (MRC-5). The toxicity of the BIM derivatives was determined through alamarBlue assay (for *T. brucei* and MRC-5) or MTT assay (for *L. major*), and the results are expressed as the concentration of the compound that reduces cell viability by 50% calculated through dose-response curves to give half maximal inhibitory concentration (IC_50_) (see Figures S7–S11).

To determine the therapeutic window, we calculated the selectivity index (SI) of each of the compounds. The SI is a ratio that measures the window between cytotoxicity in healthy cells and the antiparasitic/antiproliferative activity by dividing the cytotoxicity value (IC_50_ in MRC-5 cells) into the compounds’ half maximal inhibitory concentration in parasites (IC_50_ in *T. brucei* and *L. major*) or cancer cells (IC_50_ in HeLa and HT-29). An SI value of 1 means that the compounds are as toxic for the healthy cell line as for the parasitic and cancer cell lines. When the SI value is bigger than 1, it means that the compounds are more active against the parasitic and cancer models than in the healthy cell line, with the magnitude of SI being the dimension of the therapeutic window. On the opposite side, an SI value lower than 1 means that the compounds are more toxic in the healthy cell line than against the parasites and cancer cells. The obtained results are presented in Table 2.

Regarding the antiparasitic activity of the BIM family, prepared, derivative **2a**, substituted with the triphenylamine group, exhibited the highest activity of the series against both *T. brucei* and *L. major* with an IC_50_ of 3.21 μM and 3.30 μM, respectively. Interestingly, the cytotoxicity in normal human cells was found to be ten-fold lower (IC_50_ = 33.69 μM). Thus, compound **2a** presented an SI value of 10.50 and 10.21 for *T. brucei* and *L. major*, respectively, indicating an interesting therapeutic potential against both parasites. The unsubstituted analogue *bis*(indolyl)methane (arundine), previously reported as an inhibitor of topoisomerase I and mitochondrial F0F1-ATP synthase in *L. donavani* (species of Leishmania, also responsible for the disease leishmaniasis), displayed an IC_50_ of 1.2 μM, which is in the same range as compound **2a** in *L. major* [30,31].

Derivative **2b**, substituted with a *N,N*-dimethyl-1-naphthylamine group, showed a more modest inhibitory effect on *T. brucei* (with a IC_50_ = 15.99 μM) in comparison to compound **2a**, and very low toxicity in healthy MRC-5 cells as well as in *Leishmania major* parasites. As a result, for *T. brucei*, the SI value for **2b** was found to be 5.93, which was lower than that calculated for compound **2a**. Hence, the functionalization of the *bis*(indolyl)methane core with the *N,N*-dimethyl-1-naphthylamine group decreased the compound’s activity in general, but most notably against *L. major* (IC_50_ > 100 μM), where the compound displayed no toxicity. In contrast to compounds **2a** and **2b**, compound **2c** displayed an IC_50_ of 14.66 and 20.67 μM in the *T. brucei* and *L. major* models, respectively, while being much more toxic towards the healthy MRC-5 cells (IC_50_ = 3.25 μM), resulting in an SI value lower than 1.

Due to the anticancer activity of different BIM derivatives previously reported [9,10,11,12], the activity of compounds **2a**–**c** against HeLa (human cervical carcinoma) and HT-29 (human colorectal adenocarcinoma) cell lines was also investigated. BIM derivatives **2a** and **2c** displayed IC_50_ values in the high micromolar range for HeLa cells, together with a poor selectivity index (0.90 and 0.11, respectively). On the other hand, the toxicity of compound **2a** against the HT-29 cell line was considerably higher compared to the HeLa cancer cell line (IC_50 HT-29_ = 3.93 μM vs. IC_50 HeLa_ = 37.53 μM) and showed an SI value of 8.57. Moreover, this BIM derivative **2a** substituted with a triphenylamine group was significantly more toxic against HT-29 than its analogue arundine (IC_50 HT-29_ = > 100 μM) [41].

In silico models to predict ADMET (absorption, distribution, metabolism, excretion and toxicity) profiles are a valuable tool in medicinal chemistry research, enabling the identification of lead molecules with desired pharmaceutical properties. The ADMET lab2.0 online server [42] was used to predict relevant physicochemical, pharmacokinetic and toxicity endpoints of the synthesized BIM derivatives as well as arundine, a BIM natural product reported as an anticancer agent (Table 3). Overall, the analysis of the results revealed that all compounds satisfied Lipinski’s rule of five (MW ≤ 500, LogP ≤ 5, nHA ≤ 10, nHD ≤ 5), which is a set of criteria used as guidelines to predict the likelihood of a compound being orally bioavailable and having favorable pharmacokinetic properties. Furthermore, the predicted toxicity results indicated that among the evaluated derivatives, compound **2a** displayed the safest drug profile, with low probability of both mutagenic and carcinogenic effects and rat acute oral toxicity.

### 2.3. Fluorescence Microscopy of ***2a***

**Figure 2:**
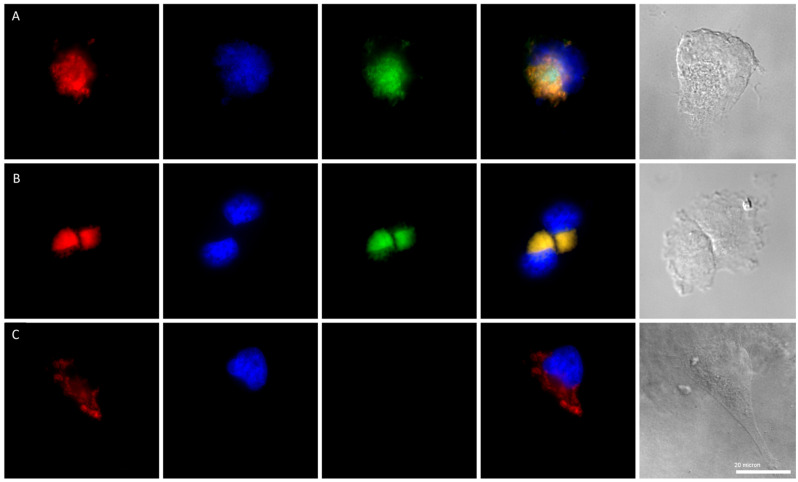
Fluorescence microscopy of MRC-5 cells treated with compound **2a**. Cells were incubated with 5 μM of compound **2a** (green channel) for 30 min (**A**) and 2 h (**B**). The mitochondria stain is represented in the red channel, and the nuclear stain is represented in the blue channel. The negative control is displayed in row (**C**). Scale bar: 20 μm.

**Figure 3:**
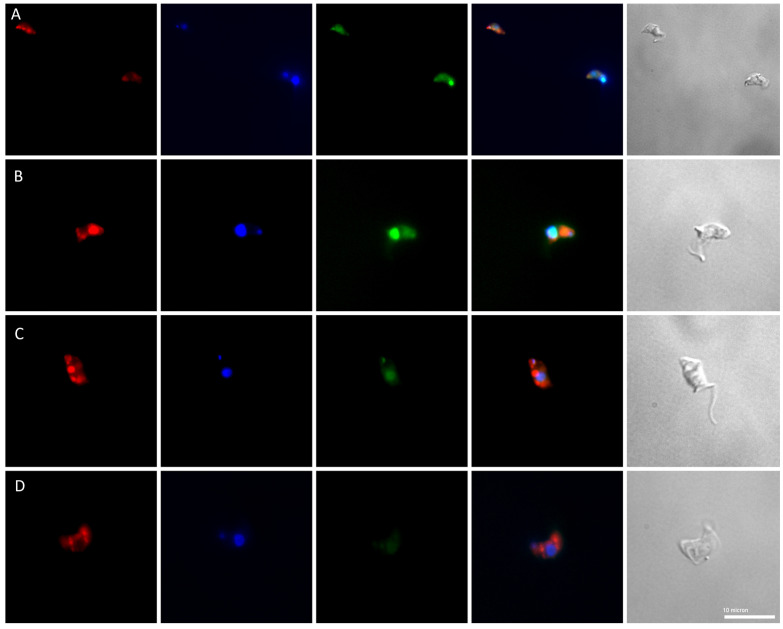
Fluorescence microscopy of parasites *T. brucei* treated with compound **2a**. Parasites were incubated with 5 μM of compound **2a** (green channel) for 30 min (**A**); 1 h (**B**); and 2 h (**C**). The mitochondria stain is represented in the red channel, and the nuclear stain is represented in the blue channel. The negative control is displayed in row (**D**). Scale bar: 10 μm.

The cellular uptake and localization of BIM derivative **2a** within MRC-5 cells and *T. brucei* parasites was studied using fluorescence microscopy, as shown in Figure 2 and Figure 3, respectively.

MRC-5 cells were incubated with 5 μM of **2a** for 30 min (Figure 2A) and 2 h (Figure 2B), and in both time points, we could detect the compound inside the cells (green channel). Moreover, the mitochondria were stained with a commercial probe (MitoTracker deep red, red channel), and as we can observe in the merged images, there is an overlap between the green and red channel fluorescence, resulting in a yellow signal. Pearson’s correlation coefficient (PCC) was used to quantify the extent of overlap between the two channels. PCC values of 0.83 ± 0.05 and 0.95 ± 0.06 were found for the overlap of compound **2a** and the MitoTracker after 30 min and 2 h of incubation, respectively. These results corroborate the strong correlation between these two probes, suggesting that compound **2a** may be accumulating in the mitochondria.

*T. brucei* parasites were also incubated with 5 μM of **2a** for 30 min (Figure 3A), 1 h (Figure 3B) and 2 h (Figure 3C), and contrary to what was observed in MRC-5 cells, the compound seems to accumulate in the nucleus over time. At 30 min, it exhibits both nuclear and cytosolic localization; however, as the incubation time increases to 1 h and 2 h, the BIM derivative (green channel) appears to overlap with the nucleus dye (blue channel). The PCC values for the overlap of compound **2a** with the nuclear dye after 30 min, 1 h and 2 h of incubation were 0.66 ± 0.19, 0.82 ± 0.08, 0.85 ± 0.08, respectively, which supports the greater accumulation of **2a** within the parasite’s nucleus as incubation time progresses.

## 3. Experimental Section

### 3.1. General

NMR spectra were obtained on a Bruker Avance III 400 at an operating frequency of 400 MHz for ^1^H and 100.6 MHz for ^13^C, using the solvent peak as an internal reference (δ relative to TMS). Peak assignments were supported by spin decoupling-double resonance and bidimensional heteronuclear techniques. High resolution mass spectra (HRMS) were obtained on an ESI/quadrupole mass spectrometer (WATERS, ACQUITY H CLASS). All reagents were purchased from Sigma-Aldrich, Acros and Fluka and used as received. Thin-layer chromatography (TLC) was carried out on 0.25 mm thick precoated silica plates (Merck Fertigplatten Kieselgel 60F_254_), and spots were visualized under ultraviolet (UV) light. Mps were determined on a Gallenkamp apparatus. Infrared spectra were recorded on a BOMEM MB 104 spectrophotometer. Fluorescence spectra were collected using a FluoroMax-4 spectrofluorometer. UV-visible absorption spectra (200–700 nm) were obtained using a Shimadzu UV/2501PC spectrophotometer. Fluorescence quantum yields were measured using 9,10-diphenylanthracene (DPA) in ethanol as standard (*ϕ_F_* = 0.95) [43]. The synthesis of BIM derivative **2a** has been reported previously [39,40].

### 3.2. Synthesis of Bis(indolyl)methanes Derivatives ***2a***–***c***

KHSO_4_ (0.56 mmol) was added to a mixture of indole (1.15 mmol) and the aldehydes **1a**–**c** (0.57 mmol) in dry methanol (3 mL), and the reaction was stirred at room temperature for 7 h [38]. Then, water (3 mL) was added to quench the reaction, and the aqueous phase was extracted with dichloromethane (3 × 20 mL). The organic phase was dried with anhydrous MgSO_4_, and the crude compounds **2a**–**c** were purified by recrystallization from dichloromethane.

#### 3.2.1. *Bis*(indolyl)methane **2a** [39,40]

Violet solid (48%); mp: 144.4–145.0 °C. ^1^H NMR (DMSO-*d*_6_) *δ* = 5.78 (s, 1H, CH) 6.84–6.87 (m, 2H, H2 and H5), 6.91–6.99 (m, 5H, H2′, H6′, H2″, H4″ and H6″), 7.02 (dt, 1H, *J* = 8.0 and 0.8 Hz, H6), 7.22 (t, 1H, *J* = 8.0 Hz, H3″ and H5″), 7.24 (d, 2H, *J* = 7.6 Hz, H3′ and H5′), 7.31 (d, 1H, *J* = 8.4 Hz, H4), 7.34 (d, 1H, *J* = 8.4 Hz, H7), 10.80 (s, 2H, 2 × NH). ^13^C NMR (DMSO-*d*_6_) *δ* = 39.0 (CH), 118.2 (C5 and C3), 119.1 (C4), 120.9 (C6), 122.5 (C2′ and C6′), 123.3 (C2″ and C6″), 123.5 (C2), 123.9 (C4″), 126.6 (C3a), 129.3 (C3′ and C5′), 129.4 (C3″ and C5″), 136.5 (C7a), 140.0 (C4′), 144.8 (C1′), 147.4 (C1″). IR (Nujol) *ν* = 3584, 3557, 3419, 3137, 2672, 2361, 2341, 1934, 1744, 1712, 1587, 1339, 1275, 1216, 1172, 1124, 1090, 1032, 1011, 935, 890, 741, 696 cm^−1^.

#### 3.2.2. *Bis*(indolyl)methane **2b**

Brown solid (80%); mp: 204.8–205.4 °C. ^1^H NMR (DMSO-*d*_6_) *δ* = 2.95 (s, 6H, 2 × CH_3_) 6.57 (s, 1H, CH), 6.73 (d, 1H, *J* = 2.4 Hz, H2), 6.84 (dt, 1H, *J* = 8.0 and 0.8 Hz, H5), 7.02 (dt, 1H, *J* = 8.0 and 0.8 Hz, H6), 7.21 (d, 2H, *J* = 7.2 Hz, H2′ and H3′), 7.26 (d, 1H, *J* = 8.0 Hz, H4), 7.34 (d, 1H, *J* = 8.4 Hz, H7), 7.47 (t, 1H, *J* = 8.2 Hz, H7′), 7.60 (t, 1H, *J* = 8.2 Hz, H6′), 8.22 (d, 1H, *J* = 8.4 Hz, H5′), 8.29 (d, 1H, *J* = 8.4 Hz, H8′), 10.79 (s, 2H, 2 × NH). ^13^C NMR (DMSO-*d*_6_) *δ* = 35.2 (CH), 45.5 (2 × CH3), 111.5 (C7), 117.6 (C5), 118.3 (C5), 119.0 (C4), 120.9 (C6), 123.3 (C5′), 124.3 (C2), 124.8 (C8′), 125.4 (C2′, C3′ and C6′) 126.0 (C7′), 126.6 (C3a), 127.4 (C4a’), 132.3 (C8a′), 136.6 (C7a). The signals of C1′ and C4′ are not visible in the ^13^C NMR. IR (Nujol) *ν* = 3583, 3223, 3188, 2676, 2360, 2336, 1622, 1602, 1231, 1216, 107, 1054, 1009, 933, 891, 872, 853, 810, 797, 782, 748 cm^−1^. HRMS (ESI) *m*/*z*: [M + H]^+^ calcd for C_29_H_26_N_3_ 416.2127, found 416.2126.

#### 3.2.3. *Bis*(indolyl)methane **2c**

Green solid (37%); mp: 207.8–208.4 °C. ^1^H NMR (DMSO-*d*_6_) *δ* = 6.19 (s, 1H, CH), 6.85 (dt, 1H, *J* = 8 and 1.2 Hz, H5), 7.01 (dd, 1H, *J* = 8.0 and 1.2 Hz, H6), 7.04 (d, 1H, *J* = 2.0 Hz, H2), 7.06 (dd, 1H, *J* = 7.6 and 1.6 Hz, H4), 7.29–7.36 (m, 4H, H7, H5′, H6′ and H7′), 7.56 (d, 1H, *J* = 8.8 Hz, H3′), 10.89 (s, 2H, 2 × NH). The signal of OH is not visible in the ^1^H NMR. IR (Nujol) *ν* = 3184, 1634, 1599, 1537, 1378, 1091, 1040, 779, 744 cm^−1^. HRMS (ESI) *m*/*z*: [M + H]^+^ calcd for C_26_H_20_N_3_O 390.1606, found 390.1593.

### 3.3. Biological Activity

#### 3.3.1. Parasite and Cell Culturing

*T. brucei* (Lister 427, antigenic type MiTat 1.2, clone 221a, bloodstream forms, “single marker” S427 (S16)) [44] were cultured at 37 °C, 5% CO_2_ in HMI-9 medium supplemented with 10% heat-inactivated fetal bovine serum (hiFBS, Invitrogen), as previously described [45].

*L. major* (MHOM/IL/80/Friedlin) promastigotes were cultured at 28 °C, 5% CO_2_ in modified RPMI-1640 medium (Invitrogen, Carlsbad, CA, USA) supplemented with 10% hiFBS [45]. Parasites were maintained in culture in their experimental growth phase (below 2 million parasites per mL for *T. brucei* and 10 million parasites per mL for *L. major*).

MRC-5 cell line (human lung fibroblast) was grown in monolayer at 37 °C, 5% CO_2_ in DMEM medium (1 g/L glucose) supplemented with 10% hiFBS, 100 U/mL penicillin, 100 mg/mL streptomycin and 2 mM L-glutamine [46].

HeLa (human cervical carcinoma) and HT-29 (human colorectal adenocarcinoma) cell lines were maintained at 37 °C, 5% CO_2_ in high glucose DMEM (4.5 g/L glucose) supplemented with 10% hiFBS, 100 U/mL penicillin, 100 mg/mL streptomycin, 2 mM L-glutamine and non-essential amino acids (1X). Cells were plated and passaged according to ATCC recommendations and were used for the experiments while in the exponential growth phase.

#### 3.3.2. Anti-Parasitic Activity

The trypanocidal activity of the compounds was assessed using the alamarBlue^®^ assay (ThermoFisher Scientific, Waltham, MA, USA) [46,47]. The stock solutions of the compounds were prepared in DMSO, and the final DMSO percentage in each well was adjusted to be less than 1%. A total of 2 × 10^4^ parasites per mL were incubated at 37 °C, 5% CO_2_ in 96-well plates (50 μL/well) alone or in the presence of an increasing concentration of compounds for 72 h. A total of 20 μL of resazurin solution (110 ng/mL) was then added to each well, and the parasites were incubated for 4 h at 37 °C. Finally, cells were lysed with 50 μL per well of SDS 3%. The plate was incubated at 37 °C for an extra hour; then, the fluorescence intensity was measured with an Infinite F200 plate reader (Tecan Austria, GmbH, Grödig, Austria), exciting at 550 nm and recording the emission at 590 nm. The results are expressed as the concentration of the compound that reduces cell growth by 50% versus untreated control cells (IC_50_). Data are presented as the average of at least three independent measurements all conducted in triplicate.

The leishmanicidal activity of the compounds was determined using MTT-based assay (Sigma-Aldrich, St. Louis, MO, USA) [48]. The stock solutions of the compounds were prepared in DMSO, and the final DMSO percentage in each well was adjusted to be less than 1%. A total of 4 × 10^6^ parasites per mL were incubated at 28 °C in 96-well plates (50 μL/well) alone or in the presence of an increasing concentration of compounds DMSO for 72 h. A total of 10 μL of MTT (5 mg/mL) was added to each well, and parasites were incubated for 4 h at 28 °C. Finally, cells were lysed with 50 μL/well of 20% SDS. The plate was incubated at 37 °C for an extra hour; then, the absorbance was measured with the Infinite F200 plate reader (TECAN Austria, GmbH, Grödig, Austria) at a wavelength of 540 nm. The IC_50_ was calculated as described above. Data are presented as the average of at least three independent measurements all conducted in triplicate.

### 3.4. Cytotoxicity

Cytotoxicity was measured in MRC-5, HeLa and HT-29 cell lines using alamarBlue^®^ assay (ThermoFisher Scientific) [46,47]. The stock solutions of the compounds were prepared in DMSO, and the final DMSO percentage in each well was adjusted to be less than 1%. A total of 5 × 10^3^ cells per mL (MRC-5, HeLa or HT-29 cells) were seeded in 96-well plates (100 μL/well) with 24 h of incubation time before compound addition at 37 °C, 5% CO_2_. Then, the compounds were added at increasing concentrations (from 0 to 100 μM), and the plates were incubated for 72 h. A total of 20 μL of resazurin solution (110 ng/mL) was added to each well, and cells were incubated for 4 h. Then, cells were lysed with 50 μL/well of 3% SDS. The plate was incubated at 37 °C for an extra hour; then, the fluorescence intensity was measured with the Infinite F200 plate reader (TECAN Austria, GmbH), exciting at 550 nm and recording the emission at 590 nm. The results are expressed as the concentration of compound that reduces cell growth by 50% versus untreated control cells (IC_50_). Data are presented as the average of at least three independent measurements all conducted in triplicate.

### 3.5. In Silico Evaluation of the Physicochemical and Pharmacokinetic Properties

The physicochemical and pharmacokinetic properties of the compounds were predicted using ADMET (absorption, distribution, metabolism, excretion and toxicity) profiles available in the ADMET lab 2.0 online server https://admetmesh.scbdd.com/ (accessed on 10 September 2023) [42].

### 3.6. Fluorescence Microscopy

MRC-5 cells (2 × 10^4^/mL) were incubated with 5 μM of compound **2a** in 0.5 mL of their respective medium for 30 min and 1 h at 37 °C and 100% humidity. Mitochondrial and nuclear staining was performed with MitoTracker deep red (200 nM) and green nuclear dye (2 drops/mL), respectively, for 30 min. Cells were washed five times with room temperature PBS and fixed with 2% paraformaldehyde for 20 min and washed two extra times with PBS. Then, the cover slides were immerged in water, and ethanol immersion of the cover slide was also necessary prior to sample processing for microscopy.

*T. Brucei* parasites (2 × 10^7^/mL) were incubated with 5 μM of compound **2a** in 0.5 mL of their respective medium for 30 min, 1 h and 2 h at 37 °C and 100% humidity. Mitochondrial and nuclear staining was performed with MitoTracker deep red (200 nM) and green nuclear dye (2 drops/mL), respectively, for 30 min. Parasites were washed five times with cold PBS and fixed with paraformaldehyde 4% for 20 min, washed two extra times with cold PBS and processed by microscope observation.

Images were acquired using a widefield Olympus ix81 microscope. Excitation was carried out with the 490 nm, 385 nm and 633 nm filters for green nuclear dye, compound **2a** and MitoTracker, respectively. Emission was detected at 512–548 nm for green nuclear dye, 420–450 nm for compound **2a** and 666–724 nm for MitoTracker. The images were processed and analyzed with Fiji software 64 bits version for windows (https://fiji.sc/, accessed on 10 September 2023).

## 4. Conclusions

In this work, we reported the synthesis of *bis*(indolyl)methane derivatives functionalized with triphenylamine, *N*,*N*-dimethyl-1-naphthylamine and 8-hydroxylquinolyl groups. The influence of the hetero(aromatic) moieties on their biological activity in the pathogenic parasites *T. brucei* and *L. major*, and as well as in the cancer cell lines HT-29 and HeLa, was investigated.

The antiparasitic activity studies revealed that the BIM substituted with triphenylamine (**2a**) displayed the best toxicity and selectivity of the series against *Trypanosoma brucei* and *Leishmania major* (with an IC_50_ of 3.21 and 3.30 μM, respectively, and ≈10-fold selectivity over healthy cell line MRC-5). Compound **2a** also showed a higher inhibitory effect against HT-29 cells than against HeLa cells, with an IC_50_ of 3.93 μM and selectivity index of 8.57. Its antiproliferative activity was found to be remarkably more active than its unsubstituted analogue, 3,3-diindolylmethane (IC_50_ > 100 μM against HT-29 cell line). Moreover, the internalization studies using fluorescence microscopy suggested that compound **2a** localizes in the mitochondria in MRC-5 cells and in the nucleus in *T. brucei* parasites. In conclusion, the *bis*(indolyl)methane derivative **2a** was demonstrated to be a potential candidate as a trypanocidal and leishmanicidal agent and as a chemotherapy drug against colon cancer.

## Figures and Tables

**Table 1:** Synthesis and photophysical data in acetonitrile solution (absorption, λ_abs_, and fluorescence emission maxima, λ_fluo_, Stokes shift, Δ_SS_ and relative fluorescence quantum yields, *ϕ_F_*) for the BIM derivatives **2a**–**c**.

Compound	Yield (%)	λ_abs_ (nm)	λ_fluo_ (nm)	Δ*_SS_*(cm^−1^)	*ϕ_F_*
**2a**	48	292	372	7365	0.28
**2b**	80	218	-	-	-
**2c**	37	241	483	20790	0.01

**Table 2:** Biological activity data for cytotoxicity in MRC-5, HeLa and HT-29 and antiparasitic activity against *T. brucei* and *L. major* represented as IC_50_ values (μM) with the error reported as *σ*. The selectivity index was calculated related to a healthy cell line (MRC-5).

Compound	IC_50_ μM					Selectivity Index (SI) *			
	*T. brucei*	*L. major*	MRC-5	HeLa	HT-29	*T. brucei*	*L. major*	HeLa	HT-29
**2a**	3.21 ± 0.04	3.30 ± 0.25	33.69 ± 3.19	37.53 ± 1.86	3.93 ± 0.49	10.50	10.21	0.90	8.57
**2b**	15.99 ± 4.44	>100	94.86 ± 7.27	89.56 ± 0.15	25.44 ± 5.23	5.93	<0.95	1.06	3.73
**2c**	14.66 ± 0.05	20.67 ± 1.71	3.25 ± 0.29	28.27 ± 5.01	71.36 ± 3.99	0.22	0.16	0.11	0.05

* SI = (IC_50_ MRC-5/IC_50_ parasite or cancer cell line).

**Table 3:** Physicochemical, pharmacokinetic and toxicity (ADMET) properties of the BIM derivatives, according to ADMET lab 2.0 software.

	Properties	Compound 2a	Compound 2b	Compound 2c	Arundine
**Physicochemical**	**MW**	489.22	415.2	389.15	246.12
	**logP**	7.363	6.249	5.281	4.185
	**TPSA (Å^2^)**	34.82	34.82	64.7	31.58
	**nHA**	3	3	4	2
	**nHD**	2	2	3	2
	**nRot**	6	4	3	2
**Medicinal chemistry**	**Lipinski**	Accepted	Accepted	Accepted	Accepted
	**PAINS**	0 alerts	0 alerts	0 alerts	0 alerts
**Absorption**	**Caco-2** **Permeability (log cm/s)**	−5.14 (Yes)	−5.14 (Yes)	−5.323 (No)	−4.852 (Yes)
	**MDCK** **Permeability (cm/s)**	4 × 10^−6^ (Yes)	7 × 10^−6^ (Yes)	5 × 10^−6^ (Yes)	9 × 10^−6^ (Yes)
**Distribution**	**VD (L/Kg)**	3.16 (Yes)	3.068 (Yes)	1.843 (Yes)	1.857 (Yes)
**Metabolism**	**CYP2C9** Substrate *	0.94 (Yes)	0.96 (Yes)	0.96 (Yes)	0.97 (Yes)
**Excretion**	**CL (mL/min/kg)**	4.598	5.843	4.475	7.19
**Toxicity**	AMES Toxicity *	0.053 (Low)	0.984 (High)	0.683 (Medium)	0.609 (Medium)
	Carcinogenicity *	0.159 (Low)	0.092 (Low)	0.053 (Low)	0.044 (Low)
	Rat Acute Oral Toxicity *	0.086(Low)	0.355 (Medium)	0.2 (Low)	0.202 (Low)

MW (molecular weight); LogP (predicted octanol/water partition coefficient): optimal 0–3; nHA (number of hydrogen acceptors): optimal 0–12; nHD (number of hydrogen donors): optimal 0–7; nRot (number of rotatable bonds): optimal 0–11; TPSA (topological polar surface area): optimal 0–140; Lipinski’s rule: MW  ≤  500, LogP ≤ 5, nHA ≤ 10, nHD ≤ 5; PAINS (pan-assay interference compounds); Caco-2 permeability: optimal > −5.15; MDCK permeability (cm/s): optimal >2 × 10^−6^ cm/s; VD (volume distribution): optimal 0.04–20 L/kg; CL (clearance): >15 “high clearance”, 5–15 “moderate clearance”, <5 “low clearance”. * Probability of being positive (0–1).

## Data Availability

Data are contained within the article and Supplementary Materials.

## Supplementary Materials

The following supporting information can be downloaded at https://www.mdpi.com/article/10.3390/molecules28237728/s1; Figure S1: ^1^H NMR spectra of BIM **2a** in DMSO-*d*_6_; Figure S2: ^13^C NMR spectra of BIM **2a** in DMSO-*d*_6_; Figure S3: ^1^H NMR spectra of BIM **2b** in DMSO-*d*_6_; Figure S4: ^13^C NMR spectra of BIM **2b** in DMSO-*d*_6_; Figure S5: ^1^H NMR spectra of BIM **2c** in DMSO-*d*_6_; Figure S6: normalized UV-visible absorption and emission spectra of BIM **2a** in ACN solution; Figure S7: dose-response curve of BIM **2a**–**c** in *T. brucei* parasites; Figure S8: dose-response curve of BIM **2a**–**c** in *L. major* parasites; Figure S9: dose-response curve of BIM **2a**–**c** in HeLa cells; Figure S10: dose-response curve of BIM **2a**–**c** in HT-29 cells; Figure S11: cell viability of *T. brucei*, *L. major*, HeLa and HT-29 cells treated with DMSO 1%.
