# Supplementary material for: Polyaromatic Bis(indolyl)methane Derivatives with Antiproliferative and Antiparasitic Activity
Source: Molecules. 2023 Nov 23;28(23):7728. doi: 10.3390/molecules28237728 (PMC10707942; doi:10.3390/molecules28237728)
Supplement: Supplementary file 1 [file molecules-28-07728-s001.zip › molecules-2690970-supplementary.pdf]

Supporting Information

Polyaromatic *bis*(indolyl)methane derivatives with antiproliferative and antiparasitic activity

Raquel C. R. Gonçalves[1,2], Pablo Peñalver[3], Susana P. G. Costa[1], Juan C. Morales[3] and Maria Manuela M. Raposo[1*]

[1]Centre of Chemistry, University of Minho, Campus of Gualtar, 4710-057 Braga, Portugal.
[2]Advanced (Magnetic) Theranostic Nanostructures Lab, International Iberian Nanotechnology Laboratory, Av. Mestre José Veiga s/n, 4715-330 Braga, Portugal.
[3]Instituto de Parasitología y Biomedicina López Neyra, CSIC, PTS Granada, Avenida del Conocimiento, 17, 18016, Armilla, Granada, Spain.

*Correspondence: mfox@quimica.uminho.pt

Figure S1. ^{1}H NMR spectra of BIM **2a** in DMSO-d_6……………………………………..2

Figure S2. ^{13}C NMR spectra of BIM **2a** in DMSO-d_6……………………………........3

Figure S3. ^{1}H NMR spectra of BIM **2b** in DMSO-d_6………………………......................4

Figure S4. ^{13}C NMR spectra of BIM **2b** in DMSO-d_6………………………......................5

Figure S5. ^{1}H NMR spectra of BIM **2c** in DMSO-d_6..……………………….......................6

Figure S6. Normalized UV-visible absorption and emission spectra of BIM **2a** in ACN solution……………………………………………………………………………………7

Figure S7. Dose-response curve of BIM **2a-c** in *T. brucei* parasites………………………..7

Figure S8. Dose-response curve of BIM **2a-c** in *L. major* parasites………………………...8

Figure S9. Dose-response curve of BIM **2a-c** in HeLa cells…………………………...8

Figure S10. Dose-response curve of BIM **2a-c** in HT-29 cells………………………………9

Figure S11. Cell viability of *T. brucei*, *L. major*, HeLa, and HT-29 cells treated with DMSO 1%...9

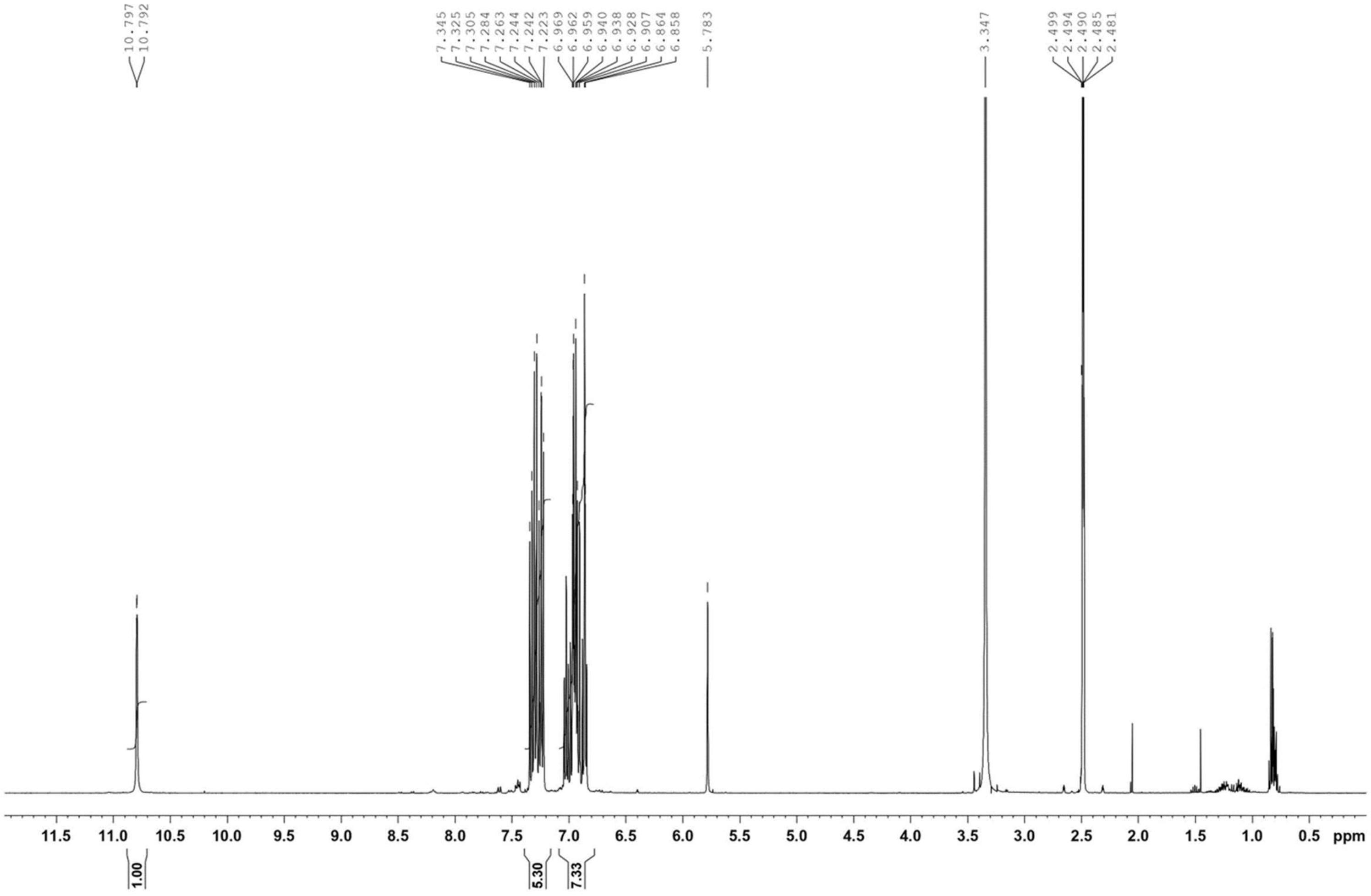

Figure S1. ^{1}H NMR spectra of BIM **2a** in DMSO-d_6.

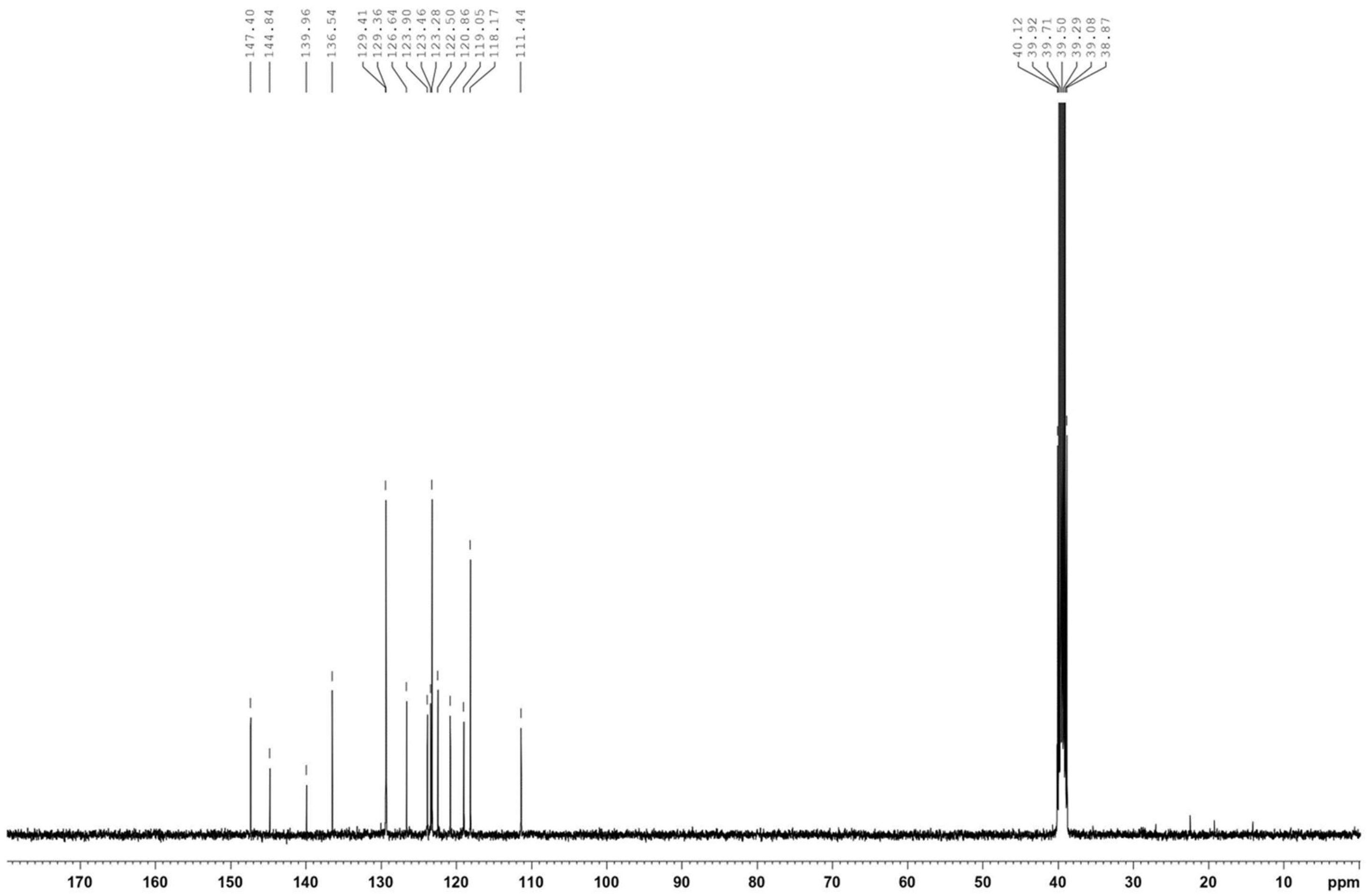

Figure S2. ^{13}C NMR spectra of BIM **2a** in DMSO-d_6.

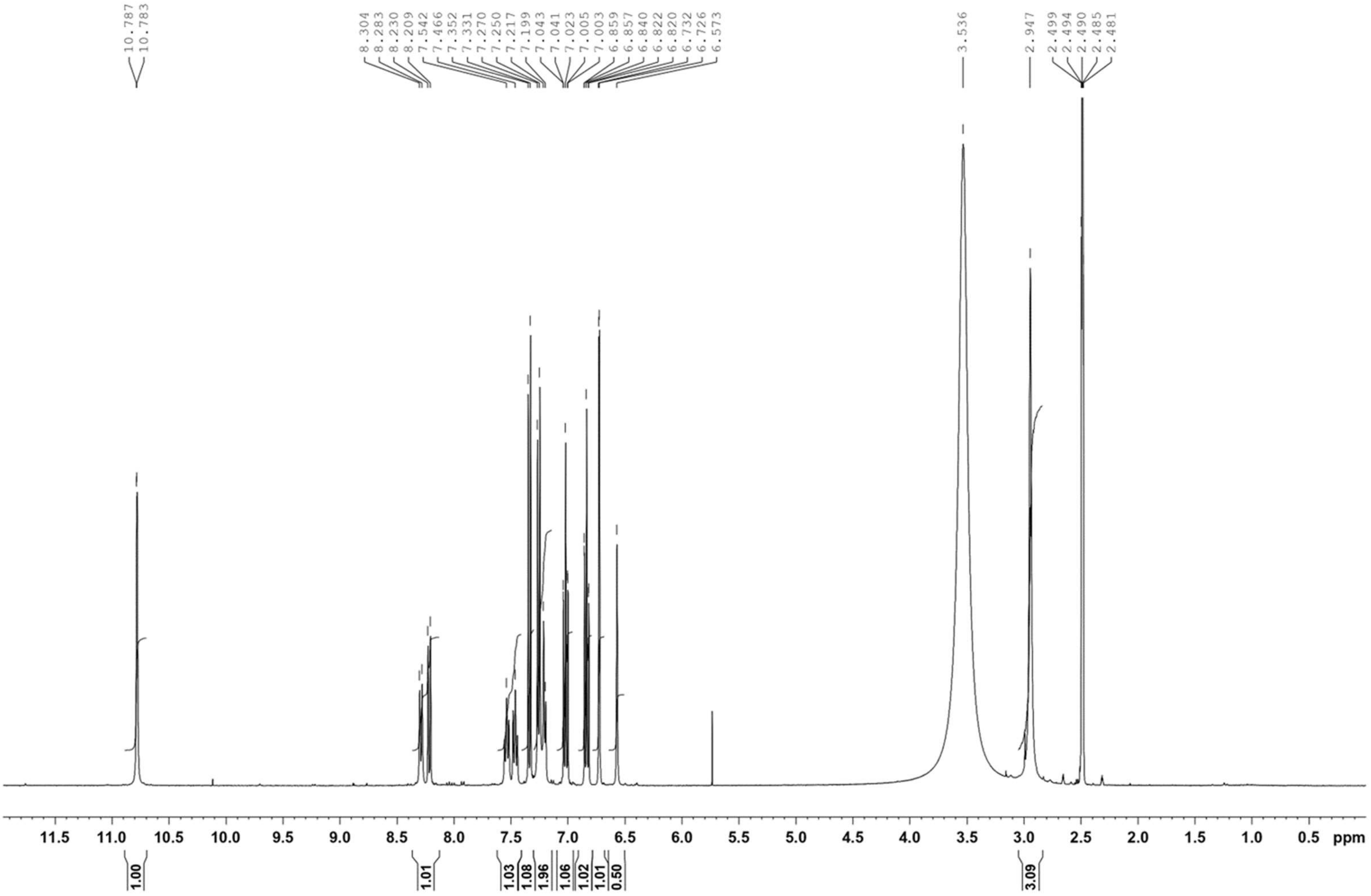

Figure S3. ^{1}H NMR spectra of BIM **2b** in DMSO-d_6.

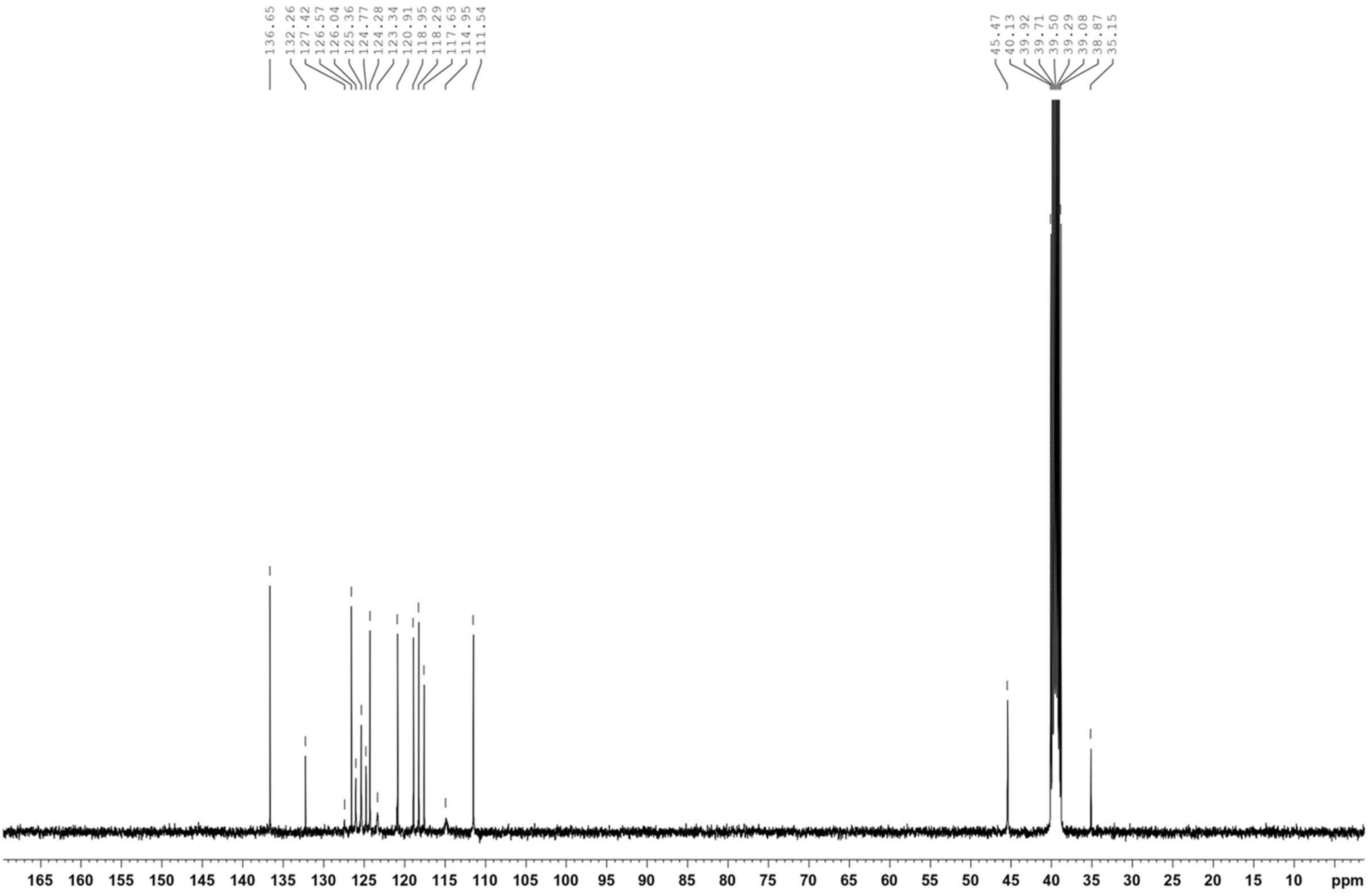

Figure S4. ^{13}C NMR spectra of BIM **2b** in DMSO-*d*$_6$.

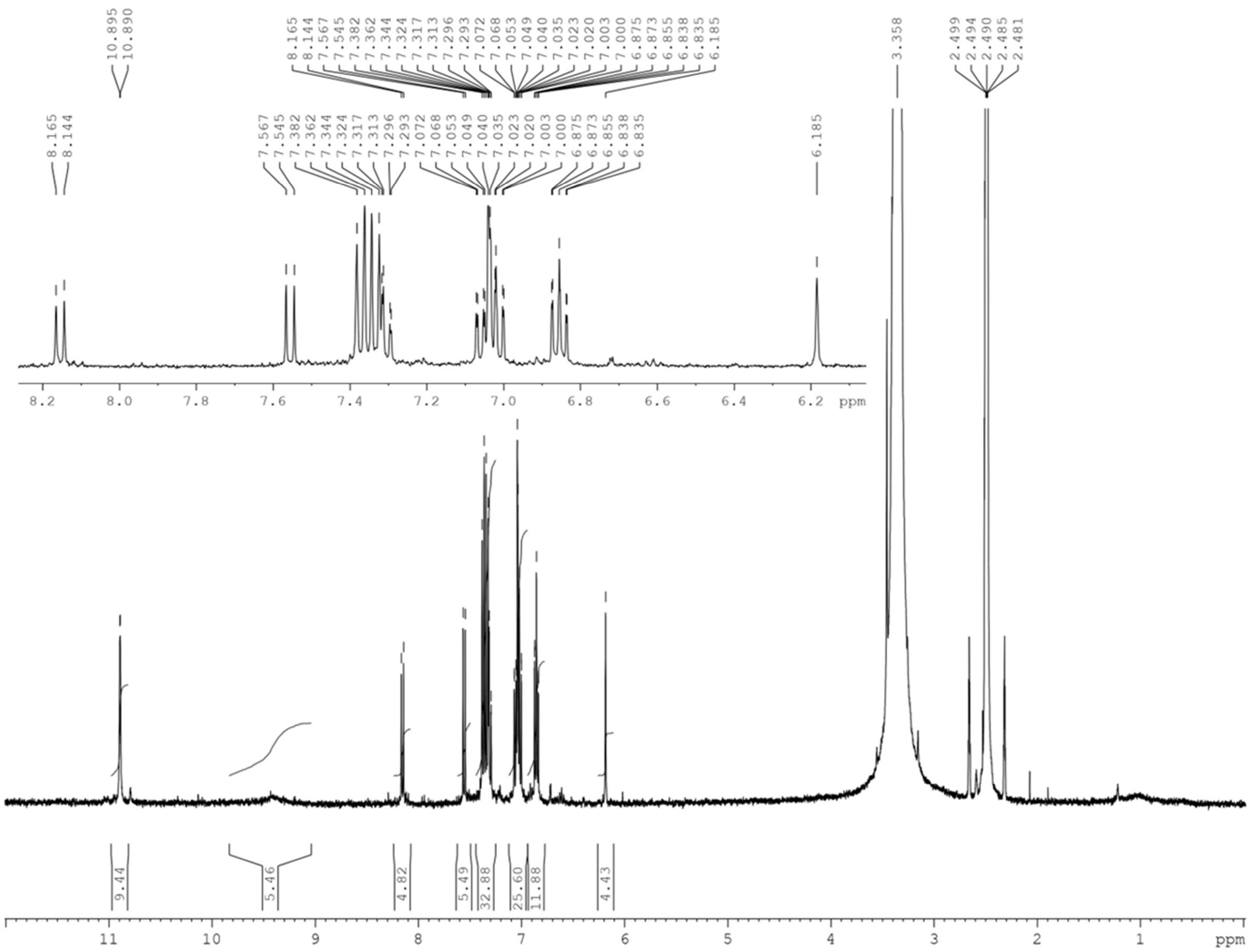

Figure S5. ^{1}H NMR spectra of BIM **2c** in DMSO-d_6.

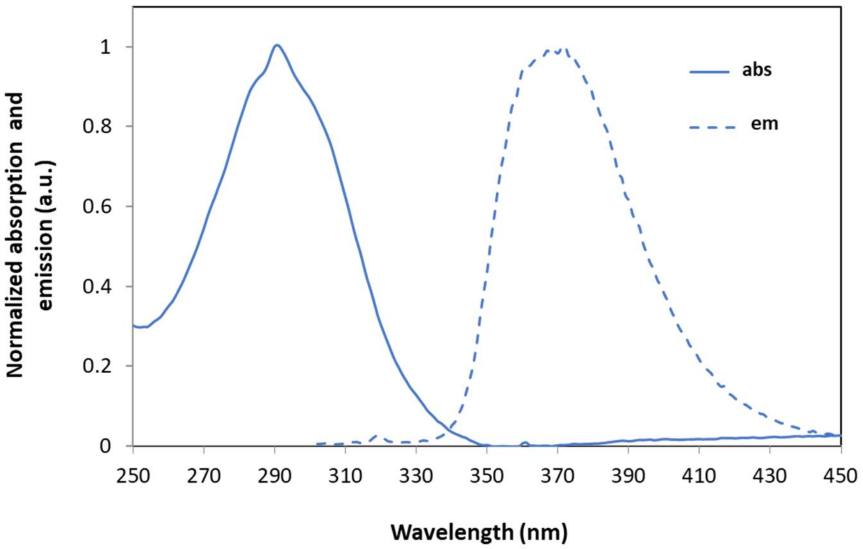

Figure S6. Normalized UV-visible absorption and emission spectra of BIM **2a** in ACN solution.

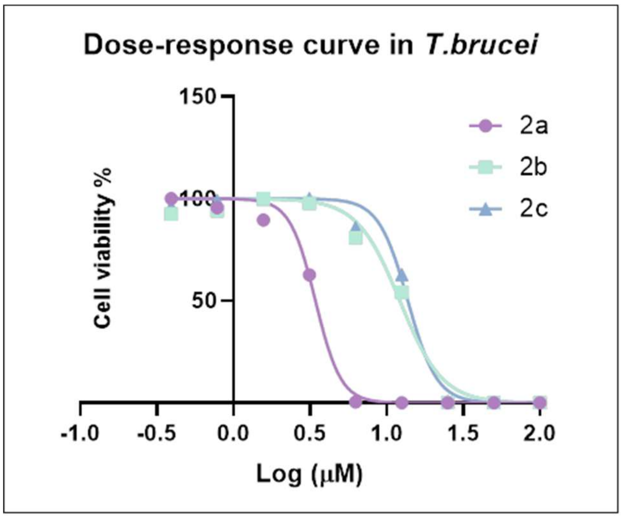

Figure S7. Dose-response curve of BIM **2a-c** in *T. brucei* parasites.

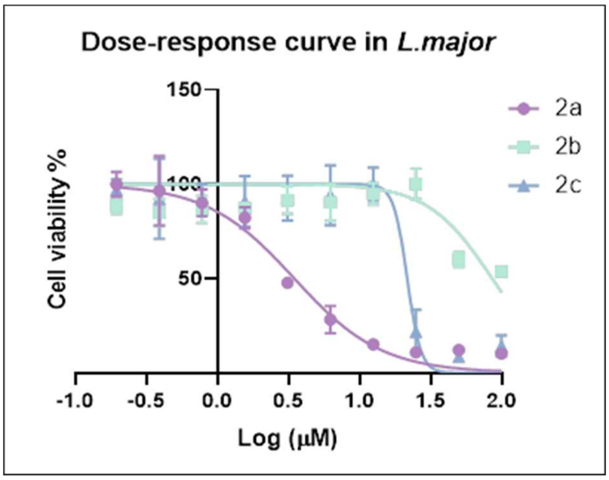

Figure S8. Dose-response curve of BIM **2a-c** in *L. major* parasites.

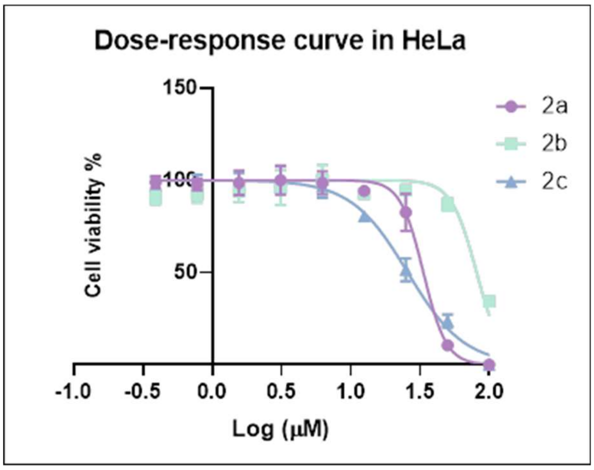

Figure S9. Dose-response curve of BIM **2a-c** in HeLa cells.

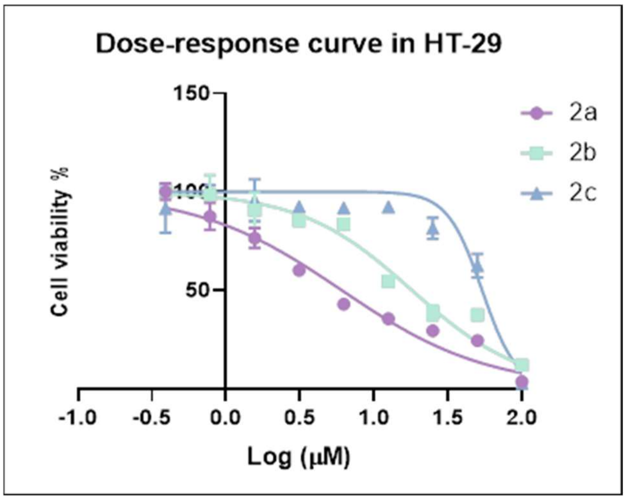

Figure S10. Dose-response curve of BIM **2a-c** in HT-29 cells.

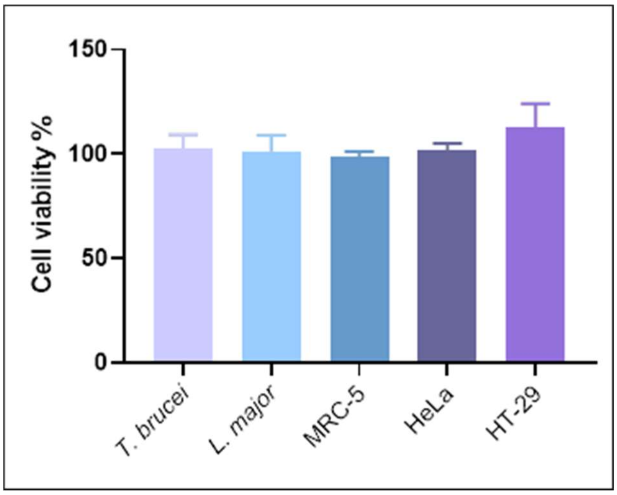

Figure S11. Cell viability of *T. brucei*, *L. major*, HeLa, and HT-29 cells treated with DMSO 1%.
